# Endocrinological Outcome of Endoscopic Transsphenoidal Surgery for Functioning and Non-Functioning Pituitary Adenoma

**DOI:** 10.21315/mjms2019.26.3.5

**Published:** 2019-06-28

**Authors:** Lee Shwu Yi, Azmi Alias, Abdul Rahman Izaini Ghani, Mohammad Badrulnizam Long Bidin

**Affiliations:** 1 Department of Neurosurgery, Hospital Kuala Lumpur, Kuala Lumpur, Malaysia; 2 Department of Neurosciences, School of Medical Sciences, Universiti Sains Malaysia, Kubang Kerian, Kelantan, Malaysia; 3 Department of Endocrinology, Hospital Kuala Lumpur, Kuala Lumpur, Malaysia

**Keywords:** endoscopy, pituitary adenoma, transsphenoidal surgery

## Abstract

**Introduction:**

The present study analysed the (i) remission and preservation of hormones, (ii) endocrinological and anatomical complications and (iii) visual improvement after endoscopic transsphenoidal surgery (ETS).

**Methods:**

The retrospective observational study of all consecutive cases of pituitary adenoma treated with ETS in Hospital Kuala Lumpur (HKL) between 2006 and 2015. Age, sex, pre- and post-operative hormone level, tumour size, and complications were noted.

**Results:**

A total of 67 patients were diagnosed with non-functioning pituitary adenoma throughout this period. Of these, 11 patients had both visual and hormonal improvement post-operation. Of the 27 patients with tumour invaded into the cavernous sinus, 13 showed an improved vision. In the adenoma patients who had impaired hormonal function before the surgery, the hormone level normalised post-surgery in 42 patients. Moreover, 39 patients were diagnosed with functioning pituitary adenoma. Ten patients recovered from acromegaly and four patients recovered from Cushing disease within seven days post-operative. Also, five patients with functioning adenoma suffered complications.

**Conclusion:**

Outcome for the preservation and hormone recovery in non-functioning pituitary adenoma group was satisfactory, with only one patient’s hormonal level worsening. No visual deterioration and mortality were detected throughout this study. A dedicated team specialised in endoscopic transsphenoidal pituitary surgery further improved the outcome of this surgical method.

## Introduction

Surgical approaches to the pituitary region have undergone numerous refinements over the last 100 years. Diseases of the pituitary gland demand a holistic and multidisciplinary approach.

Surgery is a well-established first-line treatment for a host of pituitary lesions. Surgical decompression of the pituitary gland and its stalk may lead to the recovery of hypopituitarism caused by all forms of adenoma in up to 60% of the patients. The primary goal of surgery in pituitary lesions is the maximal removal of the tumour while preserving the gland function (1).

The common post-surgery complications include cerebrospinal fluid (CSF) leakage (4.7%), meningitis (2%), and visual deterioration (2%) (2–8). The rate of post-operative hypopituitarism in other transsphenoidal tumour resections series is commonly < 20%.

## Methods

### Patient Population and Initial Evaluation

The present retrospective observational study consisted of all consecutive cases of pituitary adenoma treated with endoscopic transsphenoidal surgery (ETS) in Hospital Kuala Lumpur (HKL) between 2006 and 2015. This study was approved by the Research and Ethics committee KKM (NMRR ID: 32233).

All patients diagnosed with functional and non-functional pituitary adenoma treated with ETS were included in this study. Moreover, patients with pituitary adenoma treated with transcranial and microscopic approach and patients treated with endoscopic transsphenoidal procedure but the histopathological examination (HPE) did not report any pituitary adenoma, were excluded.

All ETSs were performed by a single consultant neurosurgeon and otorhinolaryngological (ORL) surgeon at HKL, using the standard endoscopic surgical technique described in the literature (two surgeons/four hands).

Patients were managed through a multidisciplinary approach together with neurosurgeon, endocrinologist, neuroradiologist, and ORL surgeon. Pre-operative assessment included hormonal profile and pre-operative optimisation by the endocrinologist and the anesthetist. The radiographic evaluation consisted of a magnetic resonance imaging (MRI) scan, with and without contrast, performed pre-operatively and repeated every 3–6 months during the first year and subsequently every year. The medical records were reviewed to evaluate pre- and postoperative hormonal status, pathology reports, MRI characteristics, operative notes, and clinic follow-up notes from the patient’s neurosurgeon and endocrinologist. All patients were required to have a minimum of three months of postoperative follow-up.

### Hormonal Range

All patients underwent a baseline preoperative pituitary panel and post-operative hormonal evaluation (minimum three months post-surgical follow-up) in a retrospective manner to determine the overall impact on the pituitary gland function. The normal range of hormones was set based on the local lab results.

Before the operation, all patients underwent a hormonal and clinical assessment by an endocrinologist.

The biochemical remission of acromegaly is defined as a decrease in growth hormone (GH) < 5.0 ng/mL of functioning pituitary adenomas within 12 days post-operatively (9).

Moreover, the remission of Cushing’s disease is defined as the morning (AM) cortisol level < 137 nmol/L within seven days post-operation (10). For HKL, the current practice is to measure the AM cortisol level on post-operative day 1. If the AM cortisol level is high, the test is repeated on days 3, 7, 14, and 28.

### Imaging Characteristics

The pre-operative and post-operative MRI and CT brain reports were reviewed. The formula ABC/2 was used, where A = maximum tumour diameter, B = diameter of the tumour perpendicular to A, and C = maximum height of the tumours as reported on the MRI scan. The degree of resection was calculated by measuring the residual tumour volume in the post-operative scan, which was corroborated by reviewing the surgeons’ operation report (11).

### Visual Assessment

The Humphey’s chart is used by a dedicated neuro-ophthalmologist to interpret the visual field and assess the visual acuity pre-operatively, post-operatively, at 3–6 months for the first year, and yearly thereafter.

### Complications

The complications, such as rhinological features, CSF leaks, infection, and vascular complications (bleeding), were classified according to the anatomical structures involved in the operative stages (12, 13).

### Statistical Analysis

Descriptive analysis was performed using SPSS version 20. Chi-square test was used to study the association between the study groups with respect to the gender and age of the participants, while Fisher’s exact test was used to study the association between the study groups and improvement in the vision field. A combination of chi-square test and Fisher’s exact test was used to study the correlation between the study groups and their complications.

The pre- and post-operative endocrine outcomes were tabulated in Microsoft Excel. Mc Nemar test was used to evaluate the pre- and post-operative endocrine outcomes for nonfunctioning pituitary adenoma patients.

## Results

### Descriptive Analysis

In this study, a total of 106 patients, diagnosed with pituitary adenoma and underwent transsphenoidal surgery, were included from 2006 until 2015. Of these, 67 patients were diagnosed with non-functioning pituitary adenoma, and 39 were diagnosed with functioning pituitary adenoma. The nonfunctioning pituitary group consisted of 58.2% (*n* = 39) males and 41.8% (*n* = 28) females, while the functioning pituitary group consisted of 51.3% (*n* = 20) males and 48.7% (*n* = 19) females. Thus, the majority of the participants in both groups were males. However, no significant association was detected between the study groups and gender (*P* = 0.489).

Nonetheless, a significant difference was noted in the age in both groups (*P* < 0.05). About 26.9% (*n* = 18) of non-functioning pituitary adenoma patients were 50–59 years-old, while 33.3% (*n* = 13) of the functioning pituitary adenoma patients were < 30 years-old (Table 1).

### Improvement in vision field (nonfunctioning pituitary adenoma) post-operation and preservation of hormones

In the non-functioning pituitary adenoma group, vision and hormones improved in 16.40% (*n* = 11) patients, while 83.60% (*n* = 56) did not report any changes within three months post-operation (Table 2).

### Improvement in vision field (nonfunctioning pituitary adenoma) post-operation and invasion to cavernous sinus

In the group of patients with tumour that has invaded into the cavernous sinus, vision was improved in 10.45% (*n* = 7) patients, while 29.85% (*n* = 20) did not exhibit any changes (Table 3).

### Pre-/post-operative endocrine outcomes for pituitary adenoma patients

In non-functioning pituitary adenoma group, 24 (35.81%) patients presented normal endocrine function pre-operatively. Of these, 5 (7.46%) patients experienced impaired endocrine function post-operatively, while that in the remaining 19 (28.35%) remained normal.

Moreover, of the 43 (64.18%) patients with impaired hormonal levels pre-operatively, 42 (62.69%) recovered within three months postoperatively. Only one (1.49%) patient exhibited poor condition plausibly due to the invasion to the cavernous sinus.

The functioning group consisted of 32 acromegaly patients and seven Cushing disease patients. Of these, 10 (31.25%) patients recovered from acromegaly within 12 weeks after the operation and four (57.14%) recovered from Cushing disease in the same time frame (Table 4).

### Pre-/post-operative endocrine outcomes for non-functioning pituitary adenoma patients

Hormones level were improved postoperatively. Also, statistically significant differences were detected between pre- and post-operation (Table 5).

### Hormones that improve after ETS

All the hormones were normalised after decompression surgery. This phenomenon was apparent for cortisol, thyroxine, TSH, and prolactin (Table 6).

### Complications

This study showed that there were more complications for functioning pituitary adenoma with 5.1% (*n* = 2) of the patients suffering postoperative bleeding, CSF leak 5.1% (*n* = 2), and rhinological problem 2.6% (*n* = 1) as compared to non-functioning pituitary adenoma, wherein only 4.5% (*n* = 3) of the patients suffered post-operative complications due to CSF leak (Table 7).

Hence, patients with functioning pituitary adenoma required more medical attention as compared to the non-functioning pituitary adenoma patients.

No mortality occurred within 30 days post-operation.

## Discussion

The development of endoscopic pituitary surgery represents the natural extension of endoscopic nasosinusal procedures. The potential benefits of the endoscopic technique include improved visualisation. With the use of wide-angle endoscopes 0°, 30°, 45°, and 70°, the operating surgeon has a much wider field of view and illumination as compared to that using a microscope.

This study focused on patients treated with ETS conducted by a single neurosurgeon in a 10-year-duration in order to analyse the remission and preservation of hormones, the endocrinological and anatomical complications, and the visual improvement in the same cohort treated with ETS.

The comparison of the present study with that of Karppinen (14) revealed a similar female and male ratio and age group; however, compared to the study by Marić et al. (11) in 2011, Marić et al.’s study consist of more females than males, and the functioning pituitary group was larger than the non-functioning group.

Furthermore, analysing the pre-operative position of the normal gland is a major step of the planning and surgery techniques, especially if the goal is to maintain the function of the pituitary gland. For many patients, maintaining the normal gland has higher priority as compared to the resection of the tumour (8).

Herein, of the 24 (35.81%) patients with normal endocrine function pre-operatively, 5 (7.46%) lost the function post-operatively. On the other hand, of the 43 (64.18%) patients with impairment pre-operatively, 42 (62.69%) recovered-post operatively. Only 1 (1.49%) patient’s hormone level declined post-ETS, which could be attributed to the extended invasion into the cavernous sinus. This phenomenon is improved when compared to 7.3% in Edward et al. (2).

However, 7.46% with a normal function pre-operatively, lost their function postoperatively; this was higher when compared to the 3.6% in Edward et al. (2). Among the patients who had lost the hormone function, prolactin level was altered in 3 out of 5 patients.

The functioning group consisted of 32 acromegaly patients and seven Cushing disease patients. Of these, 10 (31.25%) patients recovered from acromegaly and 4 (57.14%) recovered from Cushing disease. Thus, it could be deduced that all the hormones improved after decompression surgery (8, 15, 16, 17).

After ETS, vision improved mainly due to the decompression over the optic chiasma (18). Only 16.40% (*n* = 11) of the non-functioning pituitary adenoma patients in this study had improved or preserved vision and hormone function. None of the patients exhibited any deterioration in the hormones. Another factor that would determine the vision improvement is tumour invasion to the cavernous sinus. The vision was improved in 10.45% of the patients post-surgery while 29.85% did not present any improvement.

The complication of CSF leak in the non-functioning pituitary adenoma was 4.5% (*n* = 3). In the functioning pituitary adenoma, 5.1% (*n* = 2) patients showed CSF leak and epistaxis while rhinological problems occurred 2.6% (*n* = 1). When compared to the study by Abtin et al. (19), which was based on multiple large cohorts, the reported mortality rate for traditional surgery was < 1%. The 1%–4% reported incidences for both epistaxis and CSF leak were almost similar to the results obtained from the present study (8).

This small difference in the outcome of surgery might be attributed to patient factor (bleeding during operation) and tumour factors (invasion to cavernous sinus and tumour encasing the internal carotid artery) (7). In this study, the surgeon was not a factor as only one surgeon performed all the surgeries.

## Limitations

The data obtained were descriptive and from a single institution. In addition to the small number of patients, statistical analysis could not be performed between certain groups as this was a descriptive analysis study.

## Conclusions

Hormonal preservation and recovery are crucial for assessing the outcome of the surgery and the quality of life of the patient. With the advancement of endoscopy, MRI, and other modern technologies, the operation outcome has significantly improved with fewer post-operative complications.

In the current study, the outcome for the preservation and hormone recovery in the non-functioning pituitary adenoma group was satisfactory, with only one (1.49%) patient’s hormone worsening. Any visual deterioration and mortality were not observed throughout the study.

The present study was made possible due to well-coordinated teamwork between the neurosurgeon, endocrinologist, neuroradiologist, otorhinolaryngologist, neuropathologist, and neuroanatomist.

## Recommendations

The outcome of ETS can be improved by having:

- a dedicated team specialised in this type of surgery.
- major focus placed on the functioning group as the acromegaly and Cushing disease remission rate is low.
- an aggressive approach to achieve higher remission rate.
- benchmarking and adopting best practices from other institutions.

## Figures and Tables

**Table 1:** Demography for functioning and non-functioning pituitary adenoma patient

		Non-functioning pituitary adenoma	Functioning pituitary adenoma
Male		39 (58.2)	20 (51.3)
Female		28 (41.8)	19 (48.7)
Age (years)	< 30	7 (10.4)	13 (33.3)
	30–39	12 (17.9)	8 (20.5)
	40–49	16 (23.9)	5 (12.8)
	50–59	18 (26.9)	10 (25.6)
	> 60	14 (20.9)	3 (7.7)

* Chi-square test is used

**Table 2:** Improvement in vision field in non-functioning pituitary adenoma post-operation and preservation of hormone

	Improved	Deteriorated	No Changes
Non-functioning pituitary adenoma	16.40% (11)	0	83.60% (56)

* Fisher’s exact test

**Table 3:** Improvement in vision field (non-functioning pituitary adenoma) post-operation and invasion to cavernous sinus

Non-functioning		Invasion to Cavernous Sinus	

		Yes	No
Visual Outcome	Improved	10.45% (7)	5.97% (4)
	Deteriorated	0	0
	No changes	29.85% (20)	53.73% (36)

*P* = 0.10

* Fisher’s exact test

**Table 4:** Pre-/post-operative endocrine outcomes for pituitary adenoma patients

Tumour subtype	Normal/Normal	Impaired/Recovered	Impaired/Impaired	Impaired/Worse	Normal/Impaired	Grand total
Functioning	-	14 (35.89%)	25 (64.10%)	-	-	39 (100%)
Acromegaly	-	10 (31.25%)	22 (68.75%)	-	-	32 (100%)
Cushing disease	-	4 (57.14%)	3 (42.85%)	-	-	7 (100%)
Non-functioning	19 (28.35%)	42 (62.69%)	-	1 (1.49%)	5 (7.46%)	67 (100%)
Grand total	19 (17.92%)	56 (52.83%)	25 (23.58%)	1 (0.94%)	5 (4.72%)	106 (100%)

**Table 5:** Pre-/post-operative endocrine outcomes for non-functioning pituitary adenoma patients

Hormone pre-op/Hormone post-op	Normal	Abnormal	Total
Normal	19	5	24
Abnormal	42	1	43
Total	61	6	67

*P* < 0.05

* Mc Nemar test

**Table 6:** Hormones that improved after ETS

Condition	Improved	Normal
Cortisol level	8	57
Thyroxine level	8	57
TSH level	8	57
ACTH level	2	1
GH level	7	36
PRL level	8	53
LH level	7	49
FSH level	7	50
Testestrone level	4	23
Estradiol level	3	10

**Table 7:** Complications post-endoscopic transsphenoidal excision of pituitary adenoma

Complication	Yes/No	Study group		*P*

		Non-functioning pituitary adenoma	Functioning pituitary adenoma	
Bleeding	No	67 (100.0)	37 (94.9)	0.061*
	Yes	0 (0.0)	2 (5.1)	
CSF leak	No	64 (95.5)	37 (94.9)	0.879 *
	Yes	3 (4.5)	2 (5.1)	
Meningitis	No	67 (100.0)	39 (100.0)	n/a
Rhinological	No	67 (100.0)	38 (97.4)	0.188*
	Yes	0 (0.0)	1 (2.6)	
Visual deterioration	No	67 (100.0)	38 (97.4)	0.188*
	Yes	0 (0.0)	0 (0.0)	

* Fisher’s exact test
